# Monitoring Energy Expenditure Using a Multi-Sensor Device—Applications and Limitations of the SenseWear Armband in Athletic Populations

**DOI:** 10.3389/fphys.2017.00983

**Published:** 2017-11-30

**Authors:** Karsten Koehler, Clemens Drenowatz

**Affiliations:** ^1^Department of Nutrition and Health Sciences, University of Nebraska-Lincoln, Lincoln, NE, United States; ^2^Division of Physical Education, University of Education Upper Austria, Linz, Austria

**Keywords:** accelerometry, energy balance, high-intensity exercise, resistance exercise, measurement error

## Abstract

In order to monitor their energy requirements, athletes may desire to assess energy expenditure (EE) during training and competition. Recent technological advances and increased customer interest have created a market for wearable devices that measure physiological variables and bodily movement over prolonged time periods and convert this information into EE data. This mini-review provides an overview of the applicability of the SenseWear armband (SWA), which combines accelerometry with measurements of heat production and skin conductivity, to measure total daily energy expenditure (TDEE) and its components such as exercise energy expenditure (ExEE) in athletic populations. While the SWA has been shown to provide valid estimates of EE in the general population, validation studies in athletic populations indicate a tendency toward underestimation of ExEE particularly during high-intensity exercise (>10 METs) with an increasing underestimation as exercise intensity increases. Although limited information is available on the accuracy of the SWA during resistance exercise, high-intensity interval exercise, or mixed exercise forms, there seems to be a similar trend of underestimating high levels of ExEE. The SWA, however, is capable of detecting movement patterns and metabolic measurements even at high exercise intensities, suggesting that underestimation may result from limitations in the proprietary algorithms. In addition, the SWA has been used in the assessment of sleep quantity and quality as well as non-exercise activity thermogenesis. Overall, the SWA provides viable information and remains to be used in various clinical and athletic settings, despite the termination of its commercial sale.

## Introduction: tracking energy expenditure in athletes

One of the unique characteristics of athletes is that energy requirements of training and competition increase their total daily energy expenditure (TDEE) beyond those of the general population (Westerterp, 2013). Energy requirements can vary considerably depending on exercise type, intensity, and duration, but sustained levels of energy expenditure (EE) can be in the range of 5,000–8,000 kcal/day (Westerterp et al., 1986; Westerterp, 2001). This high energy turnover has implications not only for weight gain and weight loss practices, which are prominent in sports with weight classes, anti-gravitational sports, or aesthetic sports; it also necessitates a sufficient dietary energy intake, as sustained energy deficiency can result in long-term detriments including impaired bone health and infertility (Loucks et al., 2011). In addition, recent data suggest that athletic performance may also be impaired in energy-deprived athletes (Vanheest et al., 2013).

Because of the high energy demands and the consequences of energy deficiency, tracking EE is paramount for many athletes and their support staff. Considering that athletes expend up to 75% of their TDEE during exercise (Westerterp, 2013), quantifying energy needs during training and competition requires particular attention. The current gold-standard method for the assessment of TDEE in free-living situations is the doubly labeled water (DLW) method, which has been used in numerous athletic settings (Westerterp et al., 1986; Sjödin et al., 1994; Trappe et al., 1997; Hill and Davies, 2001, 2002; Ebine et al., 2002; Ekelund et al., 2002; Koehler et al., 2010). However, the time resolution is limited and the method does not differentiate between various components contributing to TDEE, such as exercise energy expenditure (ExEE) (Westerterp et al., 1986). Improved resolution is provided by indirect calorimetry (IC), the reference method for EE quantification in controlled laboratory settings (Haugen et al., 2007). However, despite recent methodological advances, the method remains mostly limited to research and exercise testing. Further, the requirement of a face mask hinders natural training behaviors such as fluid or food intake. Therefore, other approaches that do not interfere with training and competition practices are needed to reliably quantify EE, and particularly ExEE, in athletes.

Available methods include accelerometry, pedometry, heart-rate monitors, and self-report methods (Ndahimana and Kim, 2017). With the exception of self-report methods, which only provide subjective information and show low accuracy and reliability (Ndahimana and Kim, 2017), all of these approaches have been incorporated in activity monitors. These devices are less cost-prohibitive than DLW or IC, can be used during a wide range of activities and numerous settings, and allow for data collection over prolonged time intervals in large cohorts (Düking et al., 2016). Several such wearable devices, including the ActiGraph, Actical, RT3, ActivePAL, or GeneActiv, have been developed for research purposes, and various companies have introduced commercial physical activity trackers (e.g., Fitbit, Garmin, Jawbone, Nike). However, as these devices typically rely only on accelerometry, they provide mixed accuracy with regard to its ability to predict EE or time spent in different activities (Welk et al., 2007) and the ability to detect when devices are worn may be limited (Jaeschke et al., 2017).

## Technology of the SenseWear armband: features, functions, and modifications

**Figure 1:**
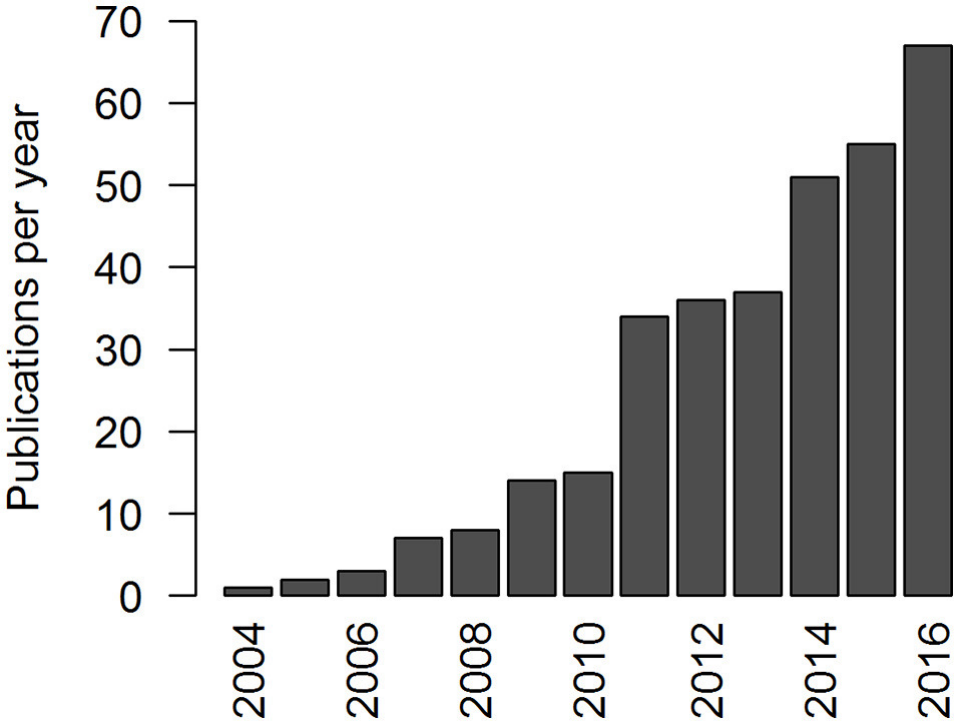
Distribution of publications including the search term “SenseWear” for the period from 2004 (first publication) to 2016 (last complete year); data source: https://www.ncbi.nlm.nih.gov/pubmed/ (Aug 14, 2017).

The SenseWear armband (SWA) developed by BodyMedia Inc. (Pittsburgh, PA, USA) combines accelerometry with additional biological variables, such as heat flux, skin temperature, near-body ambient temperature, and galvanic skin response. The device only collects data when it is in direct contact with the skin and its pattern-recognition algorithm has been shown to provide more accurate results for estimating EE and time spent in various activities when compared to the ActiGraph (Welk et al., 2007). Given these benefits, the SWA became a promising tool to objectively monitor EE in various exercise and non-exercise settings (Fruin and Rankin, 2004). Most basic principles and functions have remained the same since the initial introduction of the first prototypes in the late 1990s, but there have been several upgrades, the most notable modification being the addition of a third dimension accelerometer axis (Riou et al., 2015) along with increased data transfer and storage capacity. Per manufacturer instructions, the SWA is worn on the upper left arm, and can be used to record data continuously for up to 3–4 weeks (Koehler et al., 2013). Data can be downloaded, viewed, and exported for subsequent data processing using manufacturer software (InnerView, BodyMedia, Pittsburgh, PA). A proprietary algorithm converts raw data into estimates of EE, which are expressed both in kcal/min and metabolic equivalents (METs). In efforts to improve the validity of the SWA, this algorithm has been modified several times (Jakicic et al., 2004; Van Hoye et al., 2015). Although the technology was purchased by a competitor in 2013 and has since been discontinued (Welk et al., 2017), the SWA continues to be used extensively in research and clinical settings (Figure 1). Considering the continued popularity and the current lack of alternatives on the market, it was our goal to provide a critical review of the applicability of the SWA to measure EE specifically in athletes. As such, we provide a general overview of the strength and limitations of the SWA in the general population (section Validity of the SenseWear Armband in the General Population: Energy Expenditure, Physical Activity, and Exercise), followed by a review of the validity of the SWA in athletes and during various types of high-intensity exercise (section Validity of the SenseWear Armband during High-Intensity Exercise). We further discuss possible reasons for limitations (section Limitations of the SenseWear Armband: Algorithm vs. Methodology) and non-traditional applications of the SWA in athletic settings (section Application of the SenseWear Armband in Athletic Populations). To identify appropriate literature, a quasi-systematic PUBMED search (https://www.ncbi.nlm.nih.gov/pubmed/) was conducted in June 2017 independently by both authors, using “SenseWear” in combination with “exercise,” “activity,” or “athletes” as search terms. In addition, we included literature cited. Final inclusion was decided on by a joint decision from both authors based on each paper's relevance to the review's target group.

## Validity of the SenseWear armband in the general population: energy expenditure, physical activity, and exercise

In the general population, the SWA has been validated extensively and has been shown to provide accurate estimates of TDEE as well as EE at rest and during activities of light to moderate intensities when compared to DLW or IC (Cole et al., 2004; Fruin and Rankin, 2004; Jakicic et al., 2004; King et al., 2004; Mignault et al., 2005; Papazoglou et al., 2006; Malavolti et al., 2007; Patel et al., 2007; St-Onge et al., 2007; Johannsen et al., 2010; Casiraghi et al., 2013; Brazeau et al., 2016). When specific time periods of varying activity intensities were examined, however, the SWA generally overestimated EE at lower intensities, while EE was underestimated at higher intensities (Cole et al., 2004; Fruin and Rankin, 2004; Jakicic et al., 2004; Patel et al., 2007; Dwyer et al., 2009; Berntsen et al., 2010; Benito et al., 2012; Gastin et al., 2017). Accordingly, TDEE was overestimated in participants with low levels of TDEE and underestimated in participants with high TDEE (St-Onge et al., 2007; Johannsen et al., 2010).

It should further be considered that the accuracy of the SWA is impacted by external factors such as treadmill incline, exercise mode (e.g., running vs. bicycling), or the use of the upper vs. lower body exercise (Fruin and Rankin, 2004; Jakicic et al., 2004; Berntsen et al., 2010; Vernillo et al., 2015; Brazeau et al., 2016; Gastin et al., 2017). Specifically, underestimation of EE during uphill walking has been reported in several studies, with increasing measurement errors at steeper inclines (Fruin and Rankin, 2004; Jakicic et al., 2004; Vernillo et al., 2015). Downhill walking, on the other hand, was associated with an overestimation of EE, and—although less pronounced—measurement errors increased as declines became steeper (Vernillo et al., 2015). During stationary cycling, total EE did not differ between the SWA and IC, but individual time point data were poorly correlated: At the beginning of the cycling trial, EE was underestimated, but EE estimates by the SWA increased gradually over time even though IC values remained stable (Fruin and Rankin, 2004; Brazeau et al., 2016). Further, Gastin et al. (2017) reported an underestimation of EE during resistance type circuit exercise, most likely due to inaccuracies at higher intensities. In addition to problems related to activity type and intensity, body weight has been shown to affect measurement accuracy. Even though no particular bias toward over- or underestimation of EE was observed, measurement error increased with increasing BMI (Dwyer et al., 2009; Malavolti et al., 2012). Considering that athletes typically are on the extreme ends of the body composition spectrum (Meyer et al., 2013), it is unclear to which degree body weight or composition contribute to measurement errors in athletes.

Differences in body weight or composition may also contribute to the considerable variability of measurement accuracy at the individual level (Fruin and Rankin, 2004; Brazeau et al., 2016). Nevertheless, a recent study reported accurate measurements of TDEE with a mean difference of 2.8 kcal/day and narrow 95% confidence intervals (−34.8 to 40.3 kcal/day) and a correlation coefficient of *r* = 0.88 when comparing SWA values to DLW in 191 generally healthy adults with diverse body weight and physical activity levels (Drenowatz et al., 2017). Overall, the SWA provides valid estimates of TDEE and ExEE with a measurement error of typically <10% in a recreationally active population.

## Validity of the SenseWear armband during high-intensity exercise

To our knowledge, only one study has assessed the validity of SWA-measured TDEE specifically in athletes. Koehler et al. (2011) reported an average difference of 65 kcal/day (<2% of TDEE) between TDEE measured by SWA and DLW in 14 endurance trained athletes and a moderate to strong correlation (*r* = 0.73) However, higher levels of TDEE tended to be underestimated by the SWA, and the level of underestimation was related to the participant's exercise capacity, whereby EE was underestimated to a greater degree in better trained athletes (Koehler et al., 2011).

### Validity during high-intensity aerobic exercise

**Figure 2:**
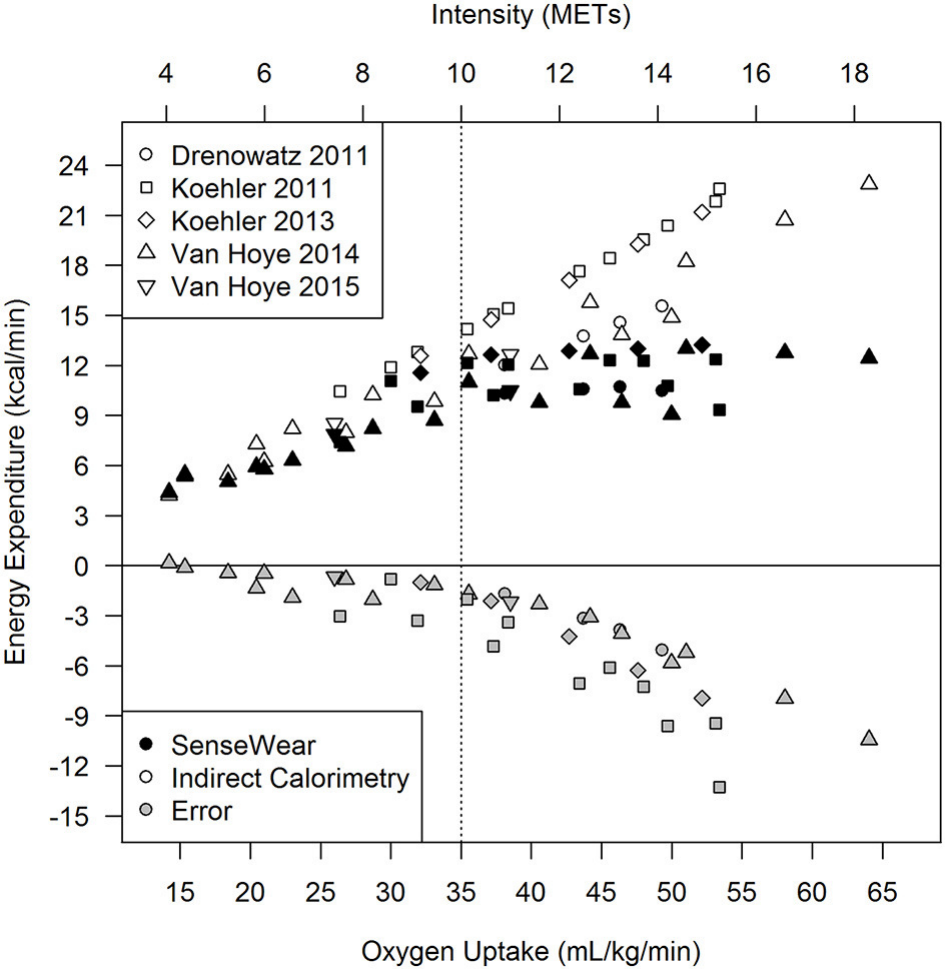
Previously published data reporting the discrepancy between energy expenditure measured with the SenseWear armband (black symbols) in comparison to the reference method (indirect calorimetry; open symbols) and the difference between SenseWear and indirect calorimetry (gray symbols). The dotted line depicts an exercise intensity of 35 mL/kg/min (10 METs). Data published by Drenowatz and Eisenmann (2011) stem from 20 male and female runners (VO_2peak_: 57 mL/kg/min); Data published by Koehler et al. (2011) stem from 14 triathletes (VO_2peak_: 58 mL/kg/min) who were assessed while running and biking; Data published by Koehler et al. (2013) stem from 19 endurance and strength trained men (VO_2peak_: 55 mL/kg/min) who were assessed while running; Data from van Hoye et al. (2014) stem from 23 male kinesiology students (VO_2peak_: 69 mL/kg/min) and 20 female kinesiology students (VO_2peak_: 53 mL/kg/min) who were assessed while walking and running; Data published by Van Hoye et al. (2015) stem from 39 male and female kinesiology students (VO_2peak_: 58 mL/kg/min) who were assessed while walking and running.

Several studies have tested the validity of the SWA during high-intensity, continuous aerobic exercise. In two independent studies in trained male athletes, the SWA underestimated ExEE during treadmill running at speeds of ~10.1 km/h (6.3 miles/h) and greater (Koehler et al., 2011, 2013). These findings were replicated by Drenowatz and Eisenmann (2011), who demonstrated that ExEE was consistently underestimated in endurance-trained athletes running at 65, 75, and 85% of their aerobic capacity, corresponding to a similar speed range (9.9–14.6 km/h; 6.2–9.1 miles/h). In another study, the SWA underestimated ExEE even at speeds from 6.0 to 7.2 km/h (3.7–4.5 miles/h) (van Hoye et al., 2014). Similar findings were also reported during stationary bicycling, whereby the SWA underestimated ExEE at workloads between 140 and 380 W (Koehler et al., 2011). In all cases, the level of underestimation increased with increasing exercise intensity (Drenowatz and Eisenmann, 2011; Koehler et al., 2011, 2013; van Hoye et al., 2014). However, visual inspection of the combined data from all five studies (Figure 2) suggests that differences between SWA and IC are rather modest at low-to-moderate exercise intensities. At exercise intensities above 35 mL/kg/min (10 METs) SWA-measured ExEE, however, tends to plateau whereas IC-measured ExEE increases continuously, resulting in a stark increase in the level of underestimation. It is noteworthy that all studies employed an incremental exercise test to assess the validity of the SWA at multiple exercise intensities. To our knowledge, only one study separately used a 30 min exercise bout at a self-selected intensity, resulting in a similar level of underestimation of 27% (Drenowatz and Eisenmann, 2011).

### Validity during resistance exercise

Only few studies have examined the accuracy of the SWA during resistance-type exercise. Benito et al. (2012) reported an underestimation of ExEE during circuit-type resistance training at 30, 50, and 70% of the 15RMmax in a mixed sample of 29 recreationally active participants. Compared to IC, SWA-estimated ExEE was 32% lower in men, corresponding to a difference of 2.3 METs, and 21% lower in women (1.1 METs). Furthermore, the degree of underestimation increased with increasing exercise intensity, although this effect was only significant in men (Benito et al., 2012). On the other hand, the SWA slightly overestimated exercise EE by an average 35 kcal per session during self-selected resistance exercise in a mixed sample of 52 participants of varying age and fitness level (Bai et al., 2016). The measurement error at the individual level was reported at 15%. However, the average exercise intensity was rather low during these sessions (3.2 METs) and may not resemble a typical resistance exercise session in athletic populations. Using a more traditional resistance training protocol of 9 exercises covering all major muscle groups with 3 sets of 10 repetitions at 70% of the 1-reptition maximum, the SWA provided accurate estimates of ExEE with an error of less than 5% and a strong correlation for ExEE (*r* = 0.77) and TDEE (*r* = 0.97) (Reeve et al., 2014). Measurement errors also remained constant across the ExEE spectrum with an almost perfect reliability of the SWA (test-retest *r* = 0.96). It should, however, be considered that ExEE was integrated over the course of the exercise bout; no information was provided on the measurement accuracy for specific exercise types (Reeve et al., 2014).

### Validity during mixed exercise forms

Similar to studies addressing resistance-type exercise, there has been only limited research examining the accuracy of the SWA during mixed exercise forms, particularly in athletic populations. Zanetti et al. (2014) assessed the accuracy of the SWA during a 42-min sport-specific intermittent exercise trial in 14 male rugby players. While there was no clear trend toward over- or underestimation of ExEE with a mean bias of −0.2 kcal/min (−1.9%), results revealed only a moderate correlation between the SWA and IC (*r* = 0.55). During a 30-min basketball-specific skill session, the SWA, however, was shown to underestimate ExEE by 1.1 kcal/min (15%) (Taylor, 2012). EE during recovery period following intermittent exercise training, on the other hand, was overestimated by 17% by the SWA when compared to IC (Zanetti et al., 2014).

## Limitations of the SenseWear armband: algorithm vs. methodology

Despite the tendency to underestimate ExEE during high-intensity exercise, available data suggest that the SWA can reliably detect activity patterns, rest periods, and varying levels of exercise intensity within individuals. For example, significant intra-individual correlations between IC and SWA was reported in 90% of endurance athletes who ran at exercise intensities between 65 and 85% VO_2max_ (Drenowatz and Eisenmann, 2011). In another study involving incremental treadmill running at speeds between 10.8 and 17.3 km/h, raw data including acceleration counts, and particularly counts in the longitudinal plane, increased continuously as workload increased (Koehler et al., 2013), demonstrating that the technology is suited to detect movement patterns even at higher exercise intensities. Consequently, limitations to the proprietary algorithm are a candidate source for the underestimation of ExEE during high-intensity exercise. Several studies have tested whether algorithm adjustments could improve the validity of the SWA during exercise. In one of the first published validation studies, Jakicic et al. (2004) reported that the accuracy of the SWA improved after algorithm revisions. After the initial algorithm underestimated ExEE during walking, stepping, and cycling by 7–29% and overestimated ExEE during arm ergometry by 29%, the researchers provided a subset of their data to develop exercise-specific proprietary equations, which reduced errors in ExEE measured by the SWA to a non-significant level. However, ExEE values, which peaked during stair stepping at 5.3–9.2 kcal/min, did not exceed the 10 MET-threshold. More recently, Van Hoye et al. (2015) compared two different algorithms during low- and moderate-intensity treadmill running in well-trained students, reasoning that a newer algorithm would provide more accurate estimates of EE as the manufacturer updates proprietary algorithms on a regular basis. When compared to the initially used algorithm (version 2.2.), data processed using a newer algorithm (version 5.2) reduced the measurement error from 18–24 to 5–17%, although ExEE remained underestimated.

## Application of the SenseWear armband in athletic populations

Despite the previously mentioned limitations, several groups have used the SWA to track EE in athletes. In adolescent sprinters undergoing high-intensity exercise training, Aerenhouts et al. (2011) measured TDEE, ExEE, and activity patterns using the SWA. When compared to self-report, the SWA registered less time spent in high-intensity activity, although this difference did not result in differences in TDEE, which was within 6% of the TDEE derived from activity diaries. The authors also highlighted the need for additional information when athletes fail to wear the SWA for 24 h. The SWA was also used to record ExEE during the competitive season in volleyball players (Woodruff and Meloche, 2013). SWA-recorded ExEE was found to be higher during games when compared to practice and warm-up sessions. Combining SWA data with diet logs and body composition assessment, the authors further concluded that the majority of the athletes were in an energy-balanced state. Using the SWA to quantify non-exercise activity thermogenesis (NEAT) among endurance athletes undergoing periods of high and low training volume, Drenowatz et al. (2013) demonstrated that the high training volume did not result in a compensatory reduction in NEAT; instead, athletes reduced their sedentary activities to allow for more training time. In professional Australian Football players, the SWA was used to document the contribution of NEAT to TDEE, which was greater on training days (85%) when compared to match days (69%) (Walker et al., 2016).

Because the SWA can be worn continuously for several days, it has also been used for the assessment of sleep quantity and quality. In male elite rugby union players, SWA-derived sleep duration was shown to be lower during game nights when compared to non-game nights, although sleep efficiency was not different (Eagles and Lovell, 2016). In another trial comparing high-intensity interval training to strength training, SWA-derived sleep efficiency was lower in the high-intensity interval condition (Kölling et al., 2016). These applications demonstrate that the SWA is well-suited to capture other biological factors, such as characteristics of sleep and NEAT, that may have important implications for athletic performance.

## Conclusion and summary

Considering that the SWA has been designed for a broad market, it is not surprising that the device tends to underestimate ExEE for periods of high-intensity exercise. Although most data has been established for aerobic exercise, the SWA seems to equally underestimate ExEE during other exercise forms. When energy expenditure is integrated over longer time periods, including rest and recovery, the measurement error becomes less pronounced and estimations of TDEE tend to be more accurate, even in athletic populations. Adjustments to the proprietary algorithm that is used to derive EE may further help to improve the validity of the SWA. Unfortunately the sale of the SWA has been terminated. Recently, a new disposable device with similar functionality has been introduced but is not available for commercial application at this time (Welk et al., 2017). Another viable option is the combination of GPS data with accelerometry and heart rate to assess EE in outdoor sports (Costa et al., 2015), although the accuracy of such devices remains to be explored. Given the current lack of alternatives, the SWA continues to be used in research and practice, emphasizing the need for the continued development of wearable devices that reliably measure EE and related variables in athletic settings.

## Author contributions

All authors listed have made a substantial, direct and intellectual contribution to the work, and approved it for publication.

### Conflict of interest statement

The authors declare that the research was conducted in the absence of any commercial or financial relationships that could be construed as a potential conflict of interest.
